# Impact of acute TTE-evidenced cardiac dysfunction on in-hospital and outpatient mortality: A multicenter NYC COVID-19 registry study

**DOI:** 10.1371/journal.pone.0283708

**Published:** 2023-03-27

**Authors:** Edwin A. Homan, Richard B. Devereux, Katherine A. Tak, Hannah W. Mitlak, Alexander Volodarskiy, Kumudha Ramasubbu, David T. Zhang, Arielle Kushman, Meridith P. Pollie, Hannah K. Agoglia, Romina Tafreshi, Parag Goyal, Leslee Shaw, Lishomwa Ndhlovu, Arindam RoyChoudhury, Evelyn Horn, Nupoor Narula, Monika M. Safford, Jonathan W. Weinsaft, Jiwon Kim

**Affiliations:** 1 Division of Cardiology, Department of Medicine, Weill Cornell Medicine / New York Presbyterian Hospital, New York, New York, United States of America; 2 New York Presbyterian Hospital–Queens, New York, New York, United States of America; 3 New York Presbyterian Hospital–Brooklyn Methodist, New York, New York, United States of America; 4 Division of General Internal Medicine, Department of Medicine, Weill Cornell Medicine / New York Presbyterian Hospital New York, New York, New York, United States of America; 5 Icahn School of Medicine at Mount Sinai, New York, New York, United States of America; 6 Division of Infectious Disease, Department of Medicine, Weill Cornell Medicine, New York, New York, United States of America; 7 Division of Biostatistics, Department of Population Health Sciences, Weill Cornell Medicine, New York, New York, United States of America; Oswaldo Cruz Foundation, BRAZIL

## Abstract

**Background:**

COVID-19 is associated with cardiac dysfunction. This study tested the relative prognostic role of left (LV), right and bi- (BiV) ventricular dysfunction on mortality in a large multicenter cohort of patients during and after acute COVID-19 hospitalization.

**Methods/Results:**

All hospitalized COVID-19 patients who underwent clinically indicated transthoracic echocardiography within 30 days of admission at four NYC hospitals between March 2020 and January 2021 were studied. Images were re-analyzed by a central core lab blinded to clinical data. Nine hundred patients were studied (28% Hispanic, 16% African-American), and LV, RV and BiV dysfunction were observed in 50%, 38% and 17%, respectively. Within the overall cohort, 194 patients had TTEs prior to COVID-19 diagnosis, among whom LV, RV, BiV dysfunction prevalence increased following acute infection (p<0.001). Cardiac dysfunction was linked to biomarker-evidenced myocardial injury, with higher prevalence of troponin elevation in patients with LV (14%), RV (16%) and BiV (21%) dysfunction compared to those with normal BiV function (8%, all p<0.05). During in- and out-patient follow-up, 290 patients died (32%), among whom 230 died in the hospital and 60 post-discharge. Unadjusted mortality risk was greatest among patients with BiV (41%), followed by RV (39%) and LV dysfunction (37%), compared to patients without dysfunction (27%, all p<0.01). In multivariable analysis, any RV dysfunction, but not LV dysfunction, was independently associated with increased mortality risk (p<0.01).

**Conclusions:**

LV, RV and BiV function declines during acute COVID-19 infection with each contributing to increased in- and out-patient mortality risk. RV dysfunction independently increases mortality risk.

## Introduction

Coronavirus disease 2019 (COVID-19) is an ongoing global pandemic that has infected hundreds of millions of people worldwide [1]. Despite the high initial mortality, better screening and therapies have improved immediate survival, resulting in a growing population at risk for subsequent clinical events [2–4]. Given that COVID-19 continues to affect an expanding global population, improved risk stratification–both acutely and after initial treatment–is of substantial importance.

Cardiac structure and function can be adversely impacted by COVID-19 through an array of processes [5–7]. COVID-induced lung disease–a common manifestation of infection–holds the potential to affect cardiovascular performance. Right ventricular function is closely coupled to pulmonary resistance, which can be altered by pulmonary parenchymal injury, hyperinflammation, and vascular thromboses [4]. Consistent with this, our group and others have shown adverse RV remodeling (dysfunction or dilation) to be common in COVID-19-infected patients and to strongly impact in-hospital prognosis [8]. Other recent studies in smaller cohorts have found right ventricular strain and RV dysfunction to identify patients with COVID-19 at high risk for poor outcomes [9, 10], and a systematic meta-analysis of 29 studies with 3813 patients found a pooled prevalence of 20% for RV dysfunction, which portended increased all-cause mortality risk [11]. It is also known that left ventricular (LV) structure and function can be affected by COVID-19 due to an an additional array of processes, including exacerbation of chronic cardiac disease, impaired myocardial perfusion due to hemodynamic instability, myocarditis and coronary thrombosis [12–14]. Prior studies have shown that COVID-19 can produce LV myocardial tissue injury paralleled by contractile dysfunction [15–20]. Overall, these studies were relatively limited in size and lacked outpatient follow-up data, preventing adequate assessment of relative impact of LV and RV dysfunction in COVID-19 infected patients after initial assessment. Prior work by our group has shown that in hospitalized patients with prior echocardiograms, LV and RV dysfunction prevalence increased following acute COVID-19 infection (35 to 48% and 12 to 31%, respectively) [21].

This study encompassed a racially and ethnically diverse multicenter registry of acute COVID-19 patients, in whom clinically indicated transthoracic echocardiography [18] was performed at four hospitals geographically distributed throughout New York City. TTEs were transferred to a centralized core lab for standardized analyses, and were blinded to patient characteristics. Study goals were to test (1) whether ventricular dysfunction varies in relation to biomarker profiles; (2) the relative prognostic impact of LV and RV, as well as biventricular (BiV) dysfunction, and [22] the prognostic impact of ventricular dysfunction on in- and out-patient mortality.

## Methods

### Study population

**Fig 1:**
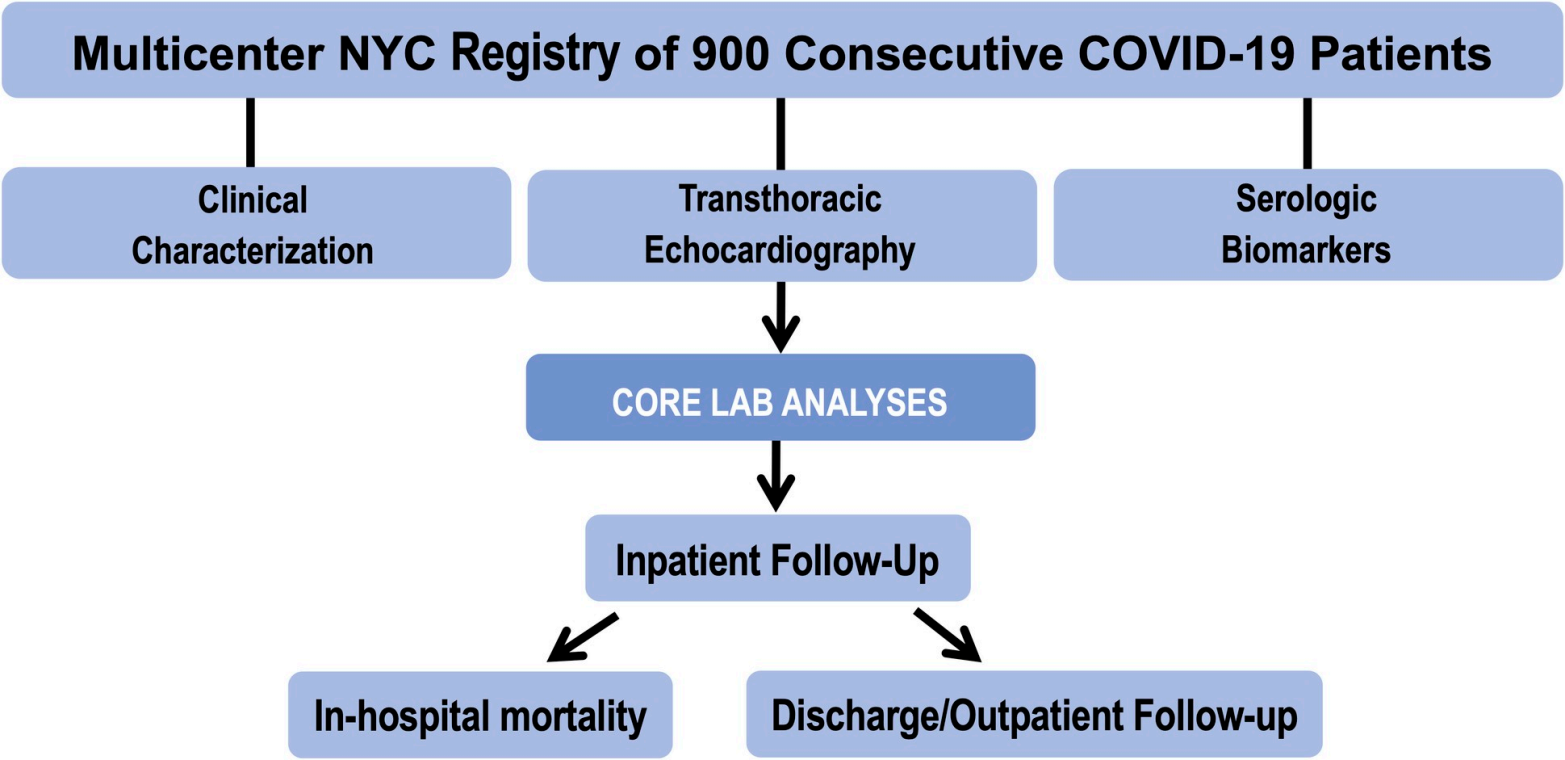
Study design. Study design entailing multicenter standardized TTE data acquisition and centralized core laboratory TTE analysis inclusive of left ventricular, right ventricular functional assessment, left atrial morphology, and hemodynamic parameters. Clinical and biomarker data were collected using a standardized electronic medical record query. Follow-up data were collected in a uniform manner to include in-hospital death, hospital discharge, and death after hospital discharge.

We reviewed records and TTEs from a study population comprised of all inpatients (age≥18 years) with COVID-19 infection (established via reverse transcriptase polymerase chain reaction testing) who underwent clinically indicated TTE between March 12, 2020, and January 4, 2021, within 30 days of admission to four hospitals in the New York-Presbyterian Hospital network (Weill Cornell Medical Center, Lower Manhattan Hospital, NYP-Queens, and NYP-Brooklyn Methodist [Fig 1]). Racial and ethnicity data was obtained from review of medical records. No patients were excluded from the study based on clinical characteristics or TTE results. For patients with multiple examinations, analyses used the initial TTE performed during hospitalization. In addition, to assess the longitudinal impact of COVID-19 on cardiac function, patients with TTE prior to COVID-19 diagnosis were also queried and these exams were retrieved for re-analyses. This research protocol was approved by the Weill Cornell Medicine Institutional Review Board, which provided approval for retrospective use of pre-existing data for research purposes without consent.

### Transthoracic echocardiography

#### Image acquisition

TTE was performed in the context of routine clinical care. The most common clinical indications for TTE during treatment for COVID-19 were dyspnea/respiratory decompensation (70%), arrhythmia (16%), elevated troponin (11%), and/or chest pain (10%). Images were acquired where possible in standard parasternal long- and short-axis views, as well as apical two-, three-, four- and five-chamber orientations and subcostal views.

#### Image analysis

TTE images were transferred to a centralized core laboratory (Weill Cornell), where data analyses were performed using an established COVID-19 registry protocol for which initial results have been previously reported [8]. In brief, dedicated analyses were performed by experienced pre-designated study investigators (J.K., R.B.D.), for whom high reproducibility of quantitative LV and RV remodeling indices have been documented [23, 24].

LV systolic function, chamber dimension, and myocardial mass were quantified based on linear dimensions measured in parasternal long-axis, with LV dysfunction defined as LVEF < 55%, consistent with established quantitative methods that have been validated in necropsy comparisons and epidemiological outcomes studies [8, 25–29]. Sex-specific binary cutoffs for LV chamber dilation and hypertrophy were derived from consensus guidelines and normative data samples [30, 31].

RV size was quantified based on end-diastolic diameter measured at the RV base in apical four-chamber orientation. RV systolic function assessment was performed using M-mode (for tricuspid annular plane excursion [TAPSE]) and tissue Doppler (for Sʹ) imaging, both of which were acquired in RV focused apical orientation. TAPSE was measured (on M-mode) as the distance of systolic excursion of the lateral tricuspid annulus along its longitudinal plane. Sʹ was measured on tissue Doppler as the peak tricuspid annular longitudinal velocity of excursion. Established cutoffs (TAPSE<1.6 cm, Sʹ<10 mm/s) were used for each parameter, and RV dysfunction was defined as presence of either abnormal TAPSE or abnormal RV Sʹ parameter [32]. Mitral and tricuspid regurgitation were graded in accordance with consensus guidelines [33]. Right ventricular systolic pressure was calculated based on tricuspid regurgitant velocity and estimated central venous pressure given the size and collapsibility of the inferior vena cava per established methods [32].

### Clinical characterization

Clinical and laboratory indices were categorized in accordance with established registry protocols [2, 8, 34–38]. Baseline data included baseline medication regimen at time of hospital admission, cardiac risk factors and subsequent initiation of COVID-19-related therapies during inpatient admission. Biomarker data included pre-specified indices known to be associated with adverse prognosis in COVID-19 infection: troponin, ferritin, C-reactive protein (CRP), D-dimer, white blood count (WBC), and hepatic transaminases. Thresholds for elevated biomarkers were defined based on site-specific laboratory thresholds at participatory hospitals. For patients with laboratory values obtained at multiple time points, peak values were used for study analyses by methodology that has been previously described by our group [2].

### Prognostic assessment

All-cause mortality was ascertained both during and after hospitalization via review of electronic medical records, blinded to echocardiographic analyses. Time to inpatient and outpatient death from any cause was calculated in relation to hospital admission and discharge dates.

#### Statistical methods

Continuous variables are reported as mean ± SD when normally distributed, and otherwise as median (interquartile range [IQR]). Normally distributed continuous indices were compared via two-tailed Student’s t-tests (for 2-group comparisons); non-normally distributed indices were compared via the Mann-Whitney U test. Categorical variables are reported as frequencies/percent and compared using the chi-square test or, if <5 expected outcomes per cell, the Fisher’s exact test. Kaplan-Meier models were used to analyze survival; patients were considered to be at risk for death following hospital admission and discharge. Fisher’s exact test and Cox proportional hazards regression models were used to evaluate univariable and multivariable associations of clinical, biomarker, and imaging parameters with mortality. Model overfitting was avoided by limiting the number of variables to 1 for every 10 outcomes. A two sided p<0.05 was considered statistically significant. Analyses were performed using SPSS version 27.0 (IBM, Armonk, New York).

## Results

### Population characteristics

The population comprised 900 consecutive COVID-19 patients in whom clinically indicated TTE was performed within 30 days of inpatient admission (median 3 days [IQR 1–9] after admission). Patients were hospitalized for a median of 13 days [IQR 6–27] with median in- and outpatient follow-up of 53 days [IQR 17–170]. Cardiac remodeling indices were measurable in the following proportion of patients: LVEF (100%), LVIDd (77%), LA volume index (58%), LA diameter (73%), RV diameter (76%), TAPSE (52%) or S’ (56%). LV systolic dysfunction was observed in 449 of 900 patients (50%), which was more common than RV dysfunction (170 of 450 patients, 38%), and four times as common as BiV dysfunction (114 of 450 patients, 17%). Due to risks of COVID-19 transmission and more limited TTEs performed early in the course of the pandemic, some TTEs lacked quantitative indices, including RV measurements in 50% and LA parameters in 27%. However, it should be noted that patients with or without quantifiable RV function cardiac parameters were similar with respect to conventional demographic indices and clinical risk factors as compared to those without (S1 Table), supporting the overall generalizability of our findings.

Clinical, hemodynamic and therapeutic treatment details stratified by LV, RV and BiV dysfunction are shown in Table 1. As shown, patients with LV dysfunction tended to be older (p = 0.05), were more likely to be male (p = 0.005). Unsurprisingly, prior clinically reported heart failure was associated with LV, RV, and BiV dysfunction on TTEs performed during COVID-19 hospitalization (all p<0.001). There was a stronger association with older age in patients with RV (p<0.001) and BiV dysfunction (p = 0.008). RV dysfunction was more closely associated with hypertension and diabetes in our cohort (p = 0.02 and p = 0.03, respectively). Regarding COVID-19-related respiratory failure there was no difference in LV, RV and BiV dysfunction frequency in patients requiring mechanical intubation.

**Table 1:** Clinical characteristics.

	Overall (n = 900)	*LV Dysfunction +(n = 449)	LV Dysfunction—(n = 451)	p	†RV Dysfunction + (n = 170)	RV Dysfunction—(n = 280)	p	‡BiV Dysfunction + (n = 114)	BiV Dysfunction—(n = 336)	p
**Demographic Indices**										
Age (years)	65.9 ± 15.9	66.9 ± 15.2	64.8 ± 16.6	0.05	70.8 ± 15.0	64.6 ± 16.8	**<0.001**	70.5 ± 15.2	65.8 ± 16.6	**0.008**
Male gender	61% (552)	66% (296)	57% (256)	**0.005**	59% (100)	59% (164)	0.96	65% (74)	57% (190)	0.12
Race				0.45			**0.046**			0.10
White	40% (361)	43% (194)	37% (167)		50% (85)	37% (103)		54% (61)	38% (127)	
African American	16% (146)	17% (76)	16% (70)		12% (21)	22% (62)		12% (14)	21% (69)	
Asian	25% (221)	22% (99)	27% (122)		22% (37)	20% (56)		18% (20)	22% (73)	
Other	16% (133)	15% (68)	17% (76)		15% (25)	18% (50)		16% (18)	17% (57)	
Not Reported	3% (24)	2% (10)	3% (14)		1% (2)	3% (7)		0.9% (1)	2% (8)	
Hawaiian Pacific Islander	0.2% (2)	0.2% (1)	0.2% (1)		0% (0)	0.4% (1)		0% (0)	0.3% (1)	
American Indian Alaska Native	0.2% (2)	0.2% (1)	0.2% (1)		0% (0)	0.4% (1)		0% (0)	0.3% (1)	
Hispanic ethnicity	28% (256)	30% (133)	27% (123)	0.44	25% (43)	28% (79)	0.50	28% (32)	27% (90)	0.79
Body surface area (m^2^)	1.9 ± 0.3	1.9 ± 0.2	1.8 ± 0.3	0.26	1.8 ± 0.2	1.9 ± 0.3	0.07	1.8 ± 0.2	1.8 ± 0.3	0.43
Heart rate (bpm)	88.1 ± 28.9	90.0 ± 21.1	86.1 ± 35.0	0.05	87.0 ± 21.4	85.1 ± 40.5	0.59	90.0 ± 22.1	84.0 ± 37.8	0.14
Systolic blood pressure (mmHg)	122.8 ± 21.1	122.2 ± 20.9	123.4 ± 21.3	0.47	125.2 ± 20.9	121.3 ± 21.2	0.11	119.6 ± 21.3	125.1 ± 20.8	**0.04**
Diastolic blood pressure (mmHg)	67.9 ± 14.4	68.7 ± 13.4	67.1 ± 15.5	0.14	67.7 ± 14.1	68.9 ± 15.5	0.50	68.2 ± 14.5	68.5 ± 15.2	0.86
**Cardiovascular Risk Factors**										
Hypertension	63% (568)	65% (290)	62% (278)	0.36	69% (117)	58% (163)	**0.02**	67% (76)	61% (204)	0.26
Diabetes mellitus	42% (382)	41% (183)	44% (199)	0.31	51% (86)	40% (112)	**0.03**	45% (51)	44% (147)	0.85
Obesity§	29% (265)	30% (132)	30% (133)	0.94	30% (51)	27% (76)	0.51	30% (33)	28% (94)	0.80
Coronary artery disease||	22% (198)	25% (110)	20% (88)	0.07	28% (48)	21% (58)	0.07	27% (31)	22% (75)	0.29
Tobacco use#	30% (273)	31% (137)	30% (136)	0.91	33% (56)	25% (71)	0.08	33% (38)	27% (89)	0.16
Heart Failure	16% (143)	21% (93)	11% (50)	**<0.001**	31% (52)	8% (22)	**<0.001**	35% (40)	10% (34)	**<0.001**
**Pulmonary Disease**										
Asthma	9% (78)	8% (37)	9% (41)	0.65	14% (23)	8% (23)	0.07	12% (14)	10% (32)	0.40
COPD	7% (60)	6% (25)	8% (35)	0.19	9% (15)	5% (14)	0.11	8% (9)	6% (20)	0.47
**Baseline CV Medications**										
ACE inhibitor/ARB	31% (282)	31% (138)	32% (144)	0.70	31% (53)	28% (78)	0.45	32% (36)	28% (95)	0.50
Statin	41% (368)	43% (194)	39% (174)	0.16	48% (82)	39% (108)	**0.04**	46% (52)	41% (138)	0.40
Beta blocker	34% (302)	37% (164)	31% (138)	0.06	43% (73)	35% (98)	0.09	48% (55)	35% (116)	**0.009**
Aspirin	27% (241)	29% (132)	24% (109)	0.08	27% (45)	25% (71)	0.79	29% (33)	25% (83)	0.37
**In Hospital Clinical Course**										
**Supplemental Oxygenation**										
Mechanical intubation	39% (353)	40% (178)	39% (175)	0.80	34% (58)	31% (88)	0.56	34% (39)	32% (107)	0.64
Intubated at TTE	32% (289)	33% (146)	32% (143)	0.80	27% (46)	24% (68)	0.51	27% (31)	25% (83)	0.60
**COVID-19 Directed Medications**										
Hydroxychloroquine	44% (400)	47% (210)	42% (190)	0.16	38% (64)	37% (103)	0.86	39% (44)	37% (123)	0.70
Steroids	42% (381)	43% (193)	42% (188)	0.69	42% (71)	40% (112)	0.71	40% (45)	41% (138)	0.76
Remdesivir	22% (194)	20% (89)	23% (105)	0.21	28% (48)	21% (58)	0.07	25% (29)	23% (77)	0.58
**TTE Indications**										
Shock	9% (85)	11% (51)	8% (34)	0.05	12% (20)	6% (16)	**0.02**	14% (16)	6% (20)	**0.006**
Dyspnea	70% (628)	73% (328)	67% (300)	**0.03**	68% (116)	63% (176)	0.25	70% (80)	63% (212)	0.17
Biomarker (troponin) elevation	11% (101)	15% (68)	7% (33)	**<0.001**	13% (22)	11% (30)	0.47	16% (18)	10% (34)	0.10
Coronary artery disease	9% (78)	8% (37)	9% (41)	0.65	9% (16)	7% (19)	0.31	8% (9)	8% (26)	0.96
Valvular disease	4% (34)	3% (14)	4% (20)	0.30	5% (8)	4% (11)	0.69	3% [22]	5% (16)	0.33
Bacteremia	10% (94)	9% (41)	12% (53)	0.20	7% (11)	13% (36)	**0.03**	6% (7)	12% (40)	0.08
Arrhythmia	16% (144)	17% (74)	16% (70)	0.69	22% (38)	15% (43)	0.06	24% (27)	16% (54)	0.07
Chest pain	10% (87)	9% (41)	10% (46)	0.59	9% (15)	11% (31)	0.45	10% (11)	10% (35)	0.82

Data presented as mean ± standard deviation or percentage (count).

*LV Dysfunction was defined as LVEF<55%.

†RV dysfunction was defined as TAPSE < 1.6 cm or RV S’ < 10 cm/s.

‡Bi-Ventricular Dysfunction was defined as the presence of RV Dysfunction and LV Dysfunction.

§Obesity was defined as BMI ≥ 30 kg/m^2^.

||Coronary artery disease was defined as history of prior MI and/or coronary revascularization.

#Tobacco use indicated current and past smoking.

### Cardiac dysfunction in relation to serologic indices

Table 2 details serologic indices for COVID-19 partitioned in relation to LV, RV and BiV dysfunction. Cardiac dysfunction was closely linked to biomarker-evidenced myocardial injury as demonstrated by higher prevalence of markedly elevated troponin elevation (5x upper limit of normal) in patients with and without LV (14% vs. 6%, p<0.001), RV (16% vs 9%, p = 0.03) and BiV dysfunction (21% vs. 8%, p<0.001). Levels of some inflammatory markers including ferritin also tended to be higher among patients with LV dysfunction (1257 vs. 926 ng/mL, p = 0.04) and BiV dysfunction (1478 vs. 824 ng/mL, p = 0.01). Marked elevation of alanine aminotransferase [39] was associated with LV, RV, and BiV dysfunction (p<0.05). Conversely, despite the above noted associations between RV dysfunction and increased troponin, nearly all systemic inflammatory markers–including D-dimer, CRP, ferritin, and WBC–were equivalent between patients with and without RV dysfunction (all p>0.05), except at the more markedly elevated levels (>5x upper limit of normal) of ferritin (48% vs. 36%, p = 0.03) and alanine aminotransferase (18% vs. 11%, p = 0.04).

**Table 2:** Serologic biomarkers stratified by patterns of ventricular dysfunction.

	Overall (n = 900)	LV Dysfunction + (n = 449)	LV Dysfunction—(n = 451)	p	RV Dysfunction + (n = 170)	RV Dysfunction—(n = 280)	p	BiV Dysfunction + (n = 114)	BiV Dysfunction—(n = 336)	p
**Laboratory Indices** *										
Troponin (ng/mL)	0.06 [0.01, 0.27]	0.10 [0.03, 0.41]	0.03 [0.0, 0.14]	**<0.001**	0.08 [0.02, 0.38]	0.03 [0.01, 0.22]	**0.02**	0.10 [0.02, 0.55]	0.03 [0.1, 0.23]	**0.009**
*> ULN*	25% (192)	33% (130)	17% (62)	**<0.001**	31% (44)	25% (59)	0.23	34% (34)	25% (67)	0.09
*>5x ULN*	10% (78)	14% (57)	6% (21)	**<0.001**	16% (23)	9% (20)	**0.03**	21% (21)	8% (22)	**<0.001**
Ferritin (ng/mL)	1078 [420, 2011]	1257 [443, 2215]	926 [404, 1911]	**0.04**	1230 [284, 2640]	814 [311, 1789]	0.09	1478 [440, 3178]	824 [302, 1799]	**0.01**
*> ULN*	83% (579)	83% (296)	83% (283)	0.89	77% (92)	79% (173)	0.62	79% (68)	78% (197)	0.82
*>5x ULN*	43% (300)	46% (165)	39% (135)	0.07	48% (57)	36% (78)	**0.03**	52% (45)	36% (90)	**0.006**
D-Dimer (ng/mL)	2459 [608, 7723]	2720 [677, 8147]	2232 [510, 7383]	0.24	1861 [460, 6470]	1970 [454, 5893]	0.88	1815 [488, 6567]	1973 [447, 6186]	0.89
*> ULN*	92% (650)	93% (340)	91% (310)	0.27	86% (106)	89% (191)	0.31	84% (75)	89% (222)	0.23
*>5x ULN*	65% (456)	66% (240)	63% (216)	0.50	57% (70)	62% (132)	0.35	58% (52)	60% (150)	0.77
CRP (mg/dL)	18 [7, 29]	18 [8, 29]	17 [5, 30]	0.56	15 [4, 24]	14 [5, 27]	0.52	16 [5, 24]	13 [5, 27]	0.96
*> ULN*	93% (635)	94% (333)	92% (302)	0.31	90% (101)	91% (198)	0.85	90% (71)	91% (228)	0.80
*>5x ULN*	81% (555)	82% (292)	80% (263)	0.44	75% (84)	78% (169)	0.61	77% (61)	77% (192)	0.90
AST (units/L)	72 [35, 161]	73 [36, 190]	71 [31, 146]	0.07	60 [28, 146]	55 [30, 146]	0.64	69 [30, 184]	55 [28, 137]	0.18
*> ULN*	75% (635)	78% (333)	73% (302)	0.12	68% (108)	71% (188)	0.55	70% (75)	70% (221)	0.91
*>5x ULN*	24% (198)	27% (114)	20% (84)	**0.03**	23% (37)	20% (54)	0.47	25% (27)	20% (64)	0.27
ALT (units/L)	59 [27, 135]	61 [29, 147]	57 [26, 126]	0.22	43 [20, 138]	49 [23, 118]	0.84	46 [25, 178]	49 [22, 109]	0.36
*> ULN*	56% (469)	58% (244)	55% (225)	0.51	46% (72)	49% (129)	0.44	47% (50)	48% (151)	0.77
*>5x ULN*	15% (124)	18% (75)	12% (49)	**0.02**	18% (29)	11% (29)	**0.04**	22% (24)	11% (34)	**0.003**
WBC (10^9^/L)	15 [10, 24]	16 [11, 25]	15 [9, 22]	**0.02**	13 [9, 21]	13 [9, 22]	0.76	12 [9, 22]	14 [9, 21]	0.82

Data presented as median [interquartile range] or percentage (count).

Laboratory indices were available as follows (data reported as % [n]): Troponin (82% [841]), Ferritin (74% [761]), D-Dimer (76% [774]), CRP (76% [773]), AST (93% [949]), ALT (92% [939]), WBC (90% [917]).

*Abnormal biomarker cutoffs defined in accordance with bioassays at participatory study sites (troponin-I >0.5 ng/mL, troponin-T >0.1 ng/mL, ferritin >274 ng/mL, D-dimer >229 mg/mL, CRP >0.9 mg/dL, AST >34 units/L, ALT >49 units/L).

### Cardiac structure and function

Table 3 details left- and right-sided TTE indices in relation to binary LV and RV functional partitions. As shown, LV end-diastolic and end-systolic dilation, as well as increased LV mass, were each associated with LV and BiV dysfunction (all p<0.01), as evidenced by larger chamber size and greater eccentric hypertrophy. Patients with LV, RV, and BiV dysfunction also had increased LA volumes (p<0.001 for all).

**Table 3:** Imaging characteristics.

	Overall (n = 900)	LV Dysfunction + (n = 449)	LV Dysfunction—(n = 451)	p	RV Dysfunction +(n = 170)	RV Dysfunction-(n = 280)	p	BiV Dysfunction + (n = 114)	BiV Dysfunction—(n = 336)	p
**Left Ventricular Function/Morphology**										
LV ejection fraction (%)	55.1 ± 14.5	46.4 ± 15.2	63.8 ± 6.0	**<0.001**	47.3 ± 17.3	58.1 ± 12.4	**<0.001**	39.6 ± 15.8	58.9 ± 11.6	**<0.001**
*Advanced LV dysfunction* *	11% (101)	23% (101)	0% (0)	**<0.001**	25% (43)	6% (18)	**<0.001**	38% (43)	5% (18)	**<0.001**
LV stroke volume (mL)	0.06 ± 0.02	0.05 ± 0.02	0.07 ± 0.02	**<0.001**	0.05 ± 0.02	0.07 ± 0.02	**<0.001**	0.05 ± 0.02	0.06 ± 0.02	**<0.001**
LV cardiac output (mL/min)	5.3 ± 2.9	4.7 ± 2.1	5.7 ± 3.3	**<0.001**	4.4 ± 2.0	5.5 ± 3.8	**0.002**	4.2 ± 2.0	5.3 ± 3.5	**0.009**
Regional wall motion abnormality	15% (117)	25% (96)	5% (21)	**<0.001**	24% (33)	10% (26)	**<0.001**	29% (27)	11% (32)	**<0.001**
LV end-diastolic volume index (mL/m^2^)	64.8 ± 24.2	75.0 ± 28.8	57.5 ± 17.0	**<0.001**	67.8 ± 29.3	63.4 ± 24.3	0.14	76.2 ± 31.8	61.6 ± 23.5	**<0.001**
*LV end-diastolic dilation* †	13% (88)	23% (65)	6% (23)	**<0.001**	19% (26)	12% (27)	**0.046**	29% (25)	10% (28)	**<0.001**
LV end-systolic volume index (mL/m^2^)	32.5 ± 22.4	48.8 ± 26.3	21.5 ± 8.6	**<0.001**	39.9 ± 27.2	28.9 ± 20.5	**<0.001**	51.5 ± 28.2	27.4 ± 19.1	**<0.001**
*LV end-systolic dilation* ‡	25% (163)	57% (153)	3% (10)	**<0.001**	34% (46)	43% (19%)	**0.002**	54% (46)	16% (43)	**<0.001**
LV myocardial mass index (g/m^2^)	85.7 ± 30.7	94.4 ± 34.9	79.5 ± 25.7	**<0.001**	89.9 ± 35.8	84.0 ± 28.0	0.10	95.8 ± 37.6	83.2 ± 28.5	**0.005**
*LV hypertrophy* §	18% (120)	27% (75)	12% (45)	**<0.001**	23% (31)	15% (35)	0.07	32% (27)	14% (39)	**<0.001**
Relative wall thickness	0.35 ± 0.19	0.32 ± 0.08	0.37 ± 0.24	**0.001**	0.35 ± 0.10	0.35 ± 0.08	0.59	0.33 ± 0.10	0.35 ± 0.08	**0.03**
**Left Atrial Morphology**										
LA volume index (mL/m^2^)	34.2 ± 17.5	38.2 ± 19.8	31.0 ± 14.7	**<0.001**	40.1 ± 21.3	32.9 ± 14.6	**0.001**	44.9 ± 23.3	32.8 ± 14.5	**<0.001**
LA diameter (cm)	3.5 ± 0.9	3.6 ± 1.0	3.5 ± 0.8	0.05	4.0 ± 1.1	3.5 ± 0.08	**<0.001**	4.0 ± 1.2	3.6 ± 0.8	**0.001**
**Right Ventricular Function/Morphology**										
RV diameter (cm)	4.0 ± 0.8	4.0 ± 0.8	3.9 ± 0.7	**0.01**	4.2 ± 0.8	3.9 ± 0.7	**<0.001**	4.2 ± 0.8	3.9 ± 0.7	**<0.001**
TAPSE (cm)	2.2 ± 4.4	2.1 ± 5.1	2.3 ± 3.7	0.63	1.7 ± 4.8	2.5 ± 4.5	0.12	1.3 ± 0.3	2.5 ± 5.2	**0.02**
S’ (cm/s)	12.9 ± 4.3	12.3 ± 4.6	13.5 ± 3.9	**<0.001**	8.8 ± 2.3	14.6 ± 3.7	**<0.001**	8.5 ± 2.1	13.7 ± 4.0	**<0.001**
RV dysfunction^#^	12.9 ± 4.3	52% (114)	25% (56)	**<0.001**	100% (170)	0% (0)	-	100% (114)	17% (56)	**<0.001**
**Hemodynamic and Valvular Indices**										
PA systolic pressure (mmHg)	40.6 ± 12.9	41.4 ± 12.3	39.9 ± 13.5	0.20	43.2 ± 13.6	38.9 ± 13.3	**0.008**	43.7 ± 12.8	39.6 ± 13.7	**0.02**
Mitral regurgitation (≥2+)	9% (68)	13% (50)	5% (18)	**<0.001**	18% (29)	5% (14)	**<0.001**	22% (24)	6% (19)	**<0.001**
Tricuspid regurgitation (≥2+)	14% (109)	17% (66)	11% (43)	**0.01**	28% (32)	10% (26)	**<0.001**	30% (31)	12% (38)	**<0.001**
Central venous pressure (mmHg)	8.1 ± 4.1	8.6 ± 4.3	7.6 ± 3.9	**0.002**	9.2 ± 4.4	7.3 ± 3.9	**<0.001**	9.5 ± 4.3	7.6 ± 4.1	**<0.001**

Data presented as mean ± standard deviation or percentage (count).

* LV ejection fraction <35%

Upper-limit normative cutoffs for LV quantitative indices defined in accordance with established literature

†LV end-diastolic volume: ♀>81.4 ml/m^2^, ♂ >88.5 ml/m^2^

‡LV end-systolic volume: ♀>34.9 ml/m^2^, ♂ >40.3 ml/m^2^

§LV myocardial mass: ♀>95 g/m^2^, ♂ >115 g/m^2^).

^#^RV dysfunction: TAPSE<1.6mm or RV S’<10cm/s

Regarding right-sided chamber remodeling, patients with LV and BiV dysfunction more commonly had increased RV chamber size (p = 0.01 and p<0.001 respectively) and RV dysfunction (p<0.001 for both). Pulmonary and central venous pressures were also higher among patients with RV or BiV dysfunction (p<0.05).

### Longitudinal echo assessment

**Fig 2:**
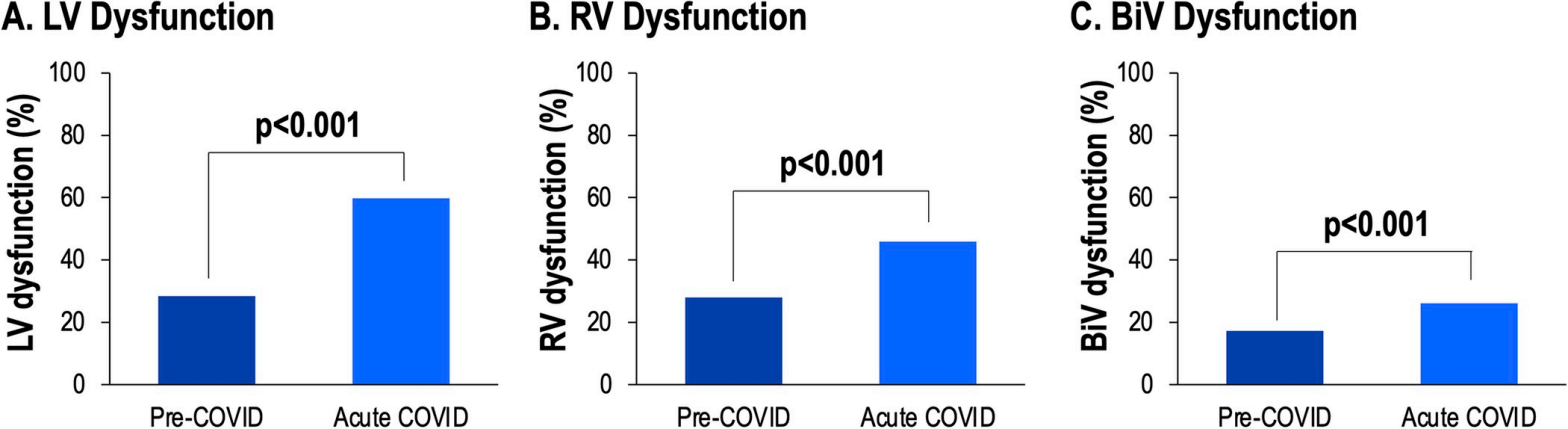
Longitudinal assessment of LV, RV and BiV function. Among patients with echo exams pre- and acutely during COVID-19 infection, prevalence of LV, RV and BiV dysfunction increased (p<0.001).

To assess the impact of COVID-19 on cardiac function longitudinally, 194 patients with TTEs prior to COVID-19 were studied (median 10 [IQR 4–22] months from acute COVID-19 TTE). In this subgroup of patients with pre-COVID-19 TTEs, LV, RV and BiV dysfunction prevalence increased following acute COVID-19 infection (28.4% vs. 59.8%, p<0.001, 27.9% vs. 45.9%; p<0.001, 17.2% vs. 26.1%; p<0.001, respectively) (Fig 2). Moreover, in this subset of patients with pre-COVID-19 exams, LVEF declined following acute COVID-19 infection (55.7±15.5% vs. 51.6±17.3%, p<0.001).

### Clinical outcomes

All-cause mortality was assessed to test the prognostic utility of cardiac function on both in-hospital as well as post-discharge clinical prognosis. Median duration of follow-up after hospital admission was 72 days (IQR 3–201) among survivors, and 72 days (IQR 26–147) in patients who died following discharge. Of the 900 patients in our cohort, a total of 290 patients died (32%) among whom 230 (79%) died in the hospital and 60 (21%) died after discharge. Unadjusted mortality risk was similarly elevated among patients with BiV dysfunction, any RV dysfunction and any LV dysfunction (41%, 39% and 37% vs. 27% without ventricular dysfunction, p<0.01 for all).

**Fig 3:**
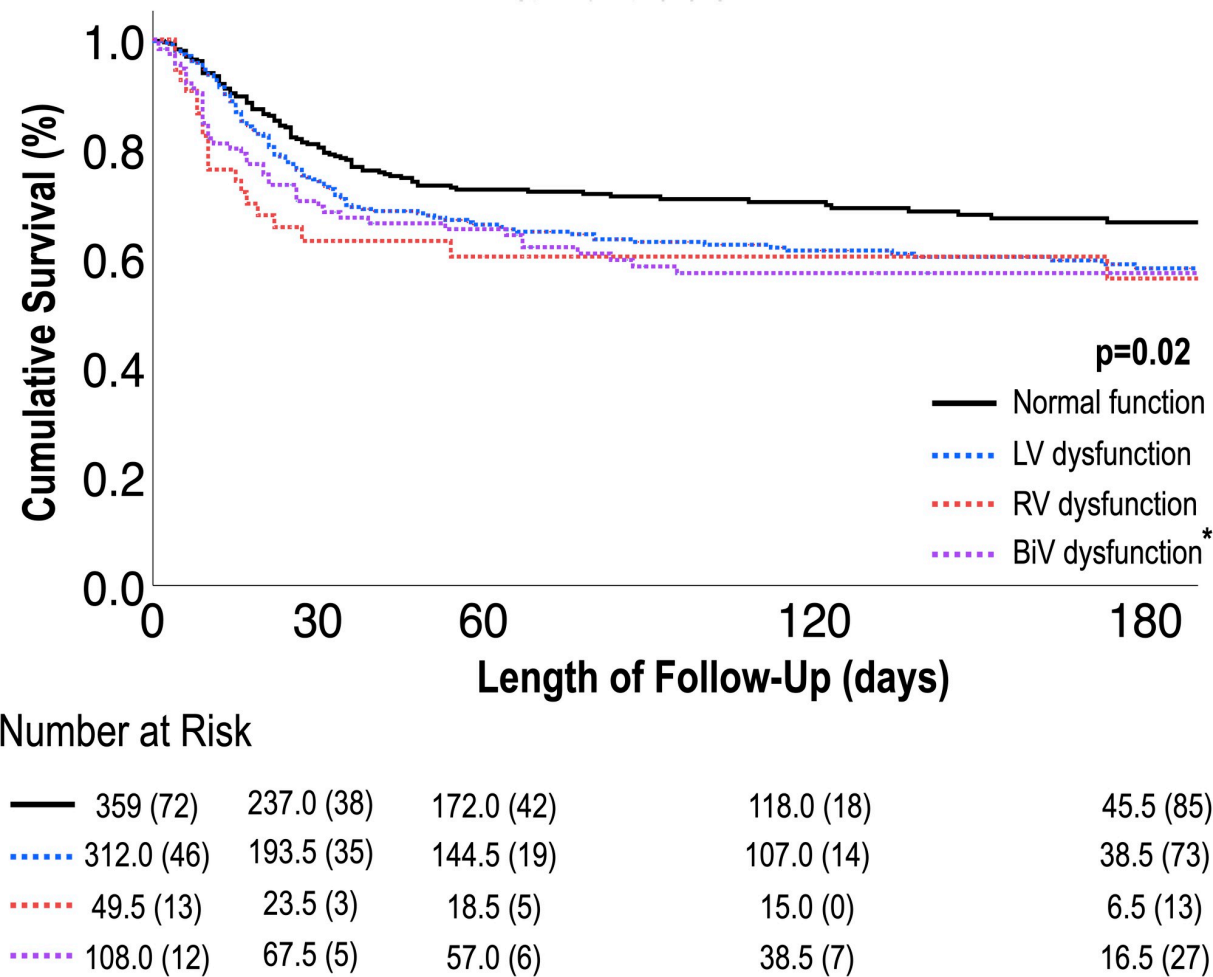
LV, RV and BiV dysfunction in relation to survival. Kaplan-Meier survival analysis for patient groups partitioned based on LV, RV and BiV dysfunction. As shown, overall mortality risk increased stepwise in relation cardiac function categorized as normal, isolated LV or RV dysfunction and BiV dysfunction, as evidenced by highest rate of death among patients with BiV dysfunction (p<0.001). [*] asterisks refer to significance when compared to patients with normal function (p<0.05).

Table 4A provides univariable Cox modeling results for clinical, biomarker and TTE-quantified imaging indices in relation to all-cause mortality. Age increased risk for death (HR 1.23 [CI 1.14–1.34] per decade; p<0.001), but other clinical risk factors were not significantly associated with mortality. There were strong associations between risk of death and log_10_-transformed serological biomarker levels: troponin (HR 1.48 [CI 1.28–1.70]), per log ng/mL; ferritin (HR 2.44 [CI 1.92–3.09]), per log ng/mL; D-dimer (HR 2.26 [CI 1.87–2.72]), per log ng/mL; and CRP (HR 3.45 [CI 2.03–5.88]), per log mg/dL (all p<0.001). Regarding imaging data, LV, RV and BiV dysfunction were each associated with increased all-cause mortality risk (HR 1.29 [CI 1.02–1.62], p = 0.03; HR 1.95 [CI 1.39–2.76], p<0.001; and HR 1.69 [CI 1.18–2.42], p = 0.004, respectively). Elevated PASP was also associated with increased mortality (HR 1.20 [CI 1.08–1.34], p<0.001). Multivariate analyses in Table 4B demonstrate that when controlling for age, LV dysfunction, and biomarkers (troponin or CRP) as in model 4, the independent predictors of mortality were age, RV dysfunction, and elevated CRP (HR 1.35 [CI 1.13–1.61], p < 0.001; HR 2.16 [CI 1.30–3.58], p = 0.003; HR 3.19 [CI 1.43–7.14], p = 0.005). Fig 3 provides Kaplan-Meier survival curves for patients stratified by LV, RV, and BiV dysfunction. As shown, there was a stepwise increase in all-cause mortality risk among patients with normal BiV function, isolated LV or RV dysfunction, and BiV dysfunction (p<0.02 for all).

**Table 4:** Predictors of all-cause mortality.

**4A. Univariable Cox Models for All-Cause Mortality**			
	**Hazard Ratio (95% CI)**		**p**
**Clinical History**			
Age (per 10 years)	1.23 [1.14–1.34]		**<0.001**
Male gender	1.04 [0.82–1.31]		0.77
Hypertension	0.99 [0.78–1.25]		0.90
Diabetes Mellitus	0.97 [0.77–1.23]		0.80
Known CAD	0.95 [0.72–1.26]		0.72
Tobacco use	0.94 [0.73–1.21]		0.61
Asthma	1.07 [0.72–1.58]		0.74
COPD	0.87 [0.53–1.42]		0.58
**Laboratory Markers** *			
Troponin	1.48 [1.28–1.70]		**<0.001**
Ferritin	2.44 [1.92–3.09]		**<0.001**
D-Dimer	2.26 [1.87–2.72]		**<0.001**
CRP	3.45 [2.03–5.88]		**<0.001**
WBC	1.07 [0.99–1.16]		0.11
**Imaging Markers**			
LV dysfunction	1.29 [1.02–1.62]		**0.03**
RV dysfunction	1.95 [1.39–2.76]		**<0.001**
BiV dysfunction	1.69 [1.18–2.42]		**0.004**
LVEF (per 10%)	0.97 [0.90–1.05]		0.44
LV end-diastolic volume (per 10 ml/m^2^)	0.97 [0.91–1.03]		0.26
*LV end-diastolic dilation*	0.77 [0.50–1.18]		0.23
LV end-systolic volume (per 10 ml/m^2^)	0.98 [0.92–1.04]		0.43
*LV end-systolic dilation*	1.14 [0.84–1.54]		0.41
LV myocardial mass (per 10 g/m^2^)	0.94 [0.90–0.99]		**0.02**
*LV hypertrophy*	0.56 [0.40–0.90]		**0.01**
PASP (per 10 mmHg)	1.20 [1.08–1.34]		**<0.001**
**4B. Multivariable Cox Models for All-Cause Mortality**			
**Model 1**		χ^2^ = 42.0, p<0.001	
Age (per 10 years)	1.27 [1.11–1.46]		**<0.001**
LV dysfunction	0.85 [0.55–1.32]		0.48
RV dysfunction	1.58 [1.05–2.38]		**0.03**
Troponin	1.66 [1.35–2.03]		**<0.001**
**Model 4**		χ^2^ = 26.7, p<0.001	
Age (per 10 years)	1.35 [1.13–1.61]		**<0.001**
LV dysfunction	0.94 [0.56–1.56]		0.80
RV dysfunction	2.16 [1.30–3.58]		**0.003**
CRP	3.19 [1.43–7.14]		**0.005**

*Analyses based on log-transformed data.

## Discussion

Our findings provide new insights with respect to predictors of adverse outcomes among a racially and ethnically diverse urban population of hospitalized patients with COVID-19 infection. Key findings of our study are as follows: First, among 900 patients with COVID-19 who underwent clinically indicated TTE across four NYC hospitals, LV, RV and BiV dysfunction were present in 50%, 38% and 17%, respectively. LV, RV, and BiV dysfunction were each strongly linked to biomarker-evidenced cardiac injury as evidenced by a nearly two-fold increased prevalence among patients with LV dysfunction compared to those without. Second, LV, RV and BiV dysfunction were linked to increased all-cause mortality risk primarily during acute hospitalization, thereby highlighting the early prognostic utility of TTE-evidenced abnormalities in acute COVID-19 hospitalization. Lastly, supporting the concept that adverse outcomes in this cohort are associated primarily with right ventricular remodeling, multivariable models which included troponin, age, and LV dysfunction showed that any RV dysfunction with or without LV dysfunction was independently associated with increased risk of all-cause death.

Our study builds on existing data examining the prognostic value of echocardiographic abnormalities in COVID-19 patients, including our own initial registry data demonstrating links between TTE-evidenced RV abnormalities and in-hospital mortality [8]. Whereas our prior study focused on the RV and its impact on mortality, smaller sample size prohibited assessment of the relative predictive utility of LV *and* RV dysfunction with short-term follow-up of only in-hospital outcomes. Study sample size also precluded examination of the prognostic impact of RV function alone, as RV remodeling inclusive of structure and function was examined as a composite variable in our prior study. Since then, in a subsequent prospective international survey of 1216 patients from 69 countries by Dweck et al., LV and RV abnormalities were reported in 39% and 33% of patients, respectively [40]. Whereas prevalence of reported cardiac dysfunction was comparable to our population, the study was limited by short timeframe (April 3 – 20, 2020) and absence of outcomes data. Several smaller studies have found LV and RV dysfunction to be associated with increased risk of death. A small retrospective study in Istanbul, Turkey stratified 90 patients into severe (defined as respiratory distress or oxygen requirement) and non-severe COVID-19 groups [41]. Patients in the severe group were older (63.3 vs 49.7 years) with larger LV and RV diameters (47.3 vs. 44.9 mm and 36.6 vs. 33.1 mm, respectively) and with impaired LV and RV function (LVEF 54 vs 62% and RV fractional change 41 vs 46%, respectively). Another multicenter study prospectively enrolled 214 hospitalized patients with COVID-19 in Denmark along with 1:1 matched controls with a median follow-up of 40 days, during which 25 patients died [10]. While these prior studies link TTE-derived cardiac impairments to short-term mortality, to our knowledge, our study is the largest to date to demonstrate the prognostic role of acute echocardiographic findings on mortality risk after COVID-19 infection with both in-hospital and post-discharge data.

Supporting the concept that RV abnormalities play an important role in COVID-19 outcomes, in an early retrospective study of 105 patients at one New York City hospital, RV enlargement was the only echocardiographic parameter independently associated with mortality (OR 4.5, [CI: 1.5–13.7]) [42]. In a UK study with partial longer term outcomes data, COVID-19 survivors were enrolled for follow-up outpatient TTE at 3 months (79 enrolled of 113 invited, out of 221 TTEs screened) [43]. Similar LV function was seen at the in-hospital baseline and post-discharge follow-up TTEs, but RV dilation and function had significantly improved during outpatient follow-up, further underscoring the point that the RV abnormalities likely represent secondary dysfunction due to acute pulmonary disease. While these studies support the premise of our present study, absence of post-discharge follow up data prohibited examination of the impact of TTE abnormalities on short- and medium-term COVID-related outcomes in the growing population of patients who survive hospitalization, which was a key focus of our present study.

The mechanism of cardiac injury and dysfunction may reflect both indirect and direct insults by the SARS-CoV2 virus. Regarding indirect insults, our observed link between myocardial dysfunction and mortality may reflect existing underlying cardiac condition that is exacerbated by systemic illness, hemodynamic compromise resulting in demand/supply mismatch and alterations in RV pre and afterload. Regarding direct insults, SARS-CoV2 is known to enter cells through interaction with angiotensin-converting enzyme 2 (ACE2), which is highly expressed in both cardiac pericytes that regulate heart microvasculature and type 2 alveolar cells in the lung [22]. Interestingly, in one gene expression analysis of endomyocardial biopsy tissue from prior patients with dilated cardiomyopathy, ACE2 was upregulated with cardiac remodeling and downregulated with reverse remodeling on beta-blocker therapy [39].

Conversely, despite the above noted association between RV dysfunction and increased troponin, nearly all systemic inflammatory markers–including D-dimer, CRP, ferritin, and WBC–were equivalent between patients with and without RV dysfunction (all p>0.05). Given the above, it is notable that we did not observe significant changes in inflammatory markers, such as CRP or D-dimer, in patients with ventricular dysfunction. Taken together, this suggests that in our cohort the ventricular dysfunction may have been driven by hemodynamic factors, hypoxia, or direct viral cardiotoxicity rather than hyperinflammatory state.

Our study had several limitations: First, our study design, despite its large scale and multicenter nature, is a retrospective TTE study among hospitalized COVID-19 patients where TTEs were only performed for clinically indicated reasons. Thus, the prevalence of cardiac dysfunction in this this cohort may be higher than that for all hospitalized patients with COVID-19, further highlighting the need for prospective evaluation of cardiac function in this population. On the other hand, while this is a select population who had clinically indicated studies, it is important to note that when examining pre-COVID TTEs, prevalence of LV and RV function increases and LVEF declines following acute COVID-19 infection, adding to the concept that while pre-existing cardiovascular disease is possible, acute functional decline during COVID-19 infection is also plausible. Furthermore, due to risks of COVID-19 transmission, particularly early on during the course of the pandemic, some TTEs were lacking quantitative indices including RV systolic measurements in 50%. However, it should be noted that patients with and without quantifiable RV dysfunction were similar with respect to conventional clinical risk factors, thereby supporting the overall generalizability of our findings. Similarly, advanced echocardiographic analysis techniques such as 2D strain were not able to be generally implemented in this real-world clinical population of TTEs, many of which lacked apical views with sufficient endocardial definition for 2D strain analysis.

## Conclusion

Our findings demonstrate LV, RV and BiV dysfunction to each decline during acute COVID-19 infection and contribute to increased in- and out-patient mortality risk. In multivariable analysis, any RV dysfunction, but not LV dysfunction, was independently associated with increased mortality. We therefore highlight the prognostic utility of TTE-derived cardiac function and underscoring the importance of screening and surveillance among patients hospitalized with COVID-19 infection. Further studies are warranted to elucidate the underlying mechanisms and long-term clinical implications of COVID-associated LV and RV dysfunction.

## Supporting information

S1 Table (DOCX)

## Abbreviations

CI: confidence interval
TTE: transthoracic echocardiography
ICU: intensive care unit
IQR: interquartile range
LV: left ventricle
LVEF: left ventricular ejection fraction
PASP: pulmonary artery systolic pressure
RV: right ventricle
TAPSE: tricuspid annular plane excursion
ULN: upper limit normal
