# Supplementary material for: Impact of acute TTE-evidenced cardiac dysfunction on in-hospital and outpatient mortality: A multicenter NYC COVID-19 registry study
Source: PLoS One. 2023 Mar 27;18(3):e0283708. doi: 10.1371/journal.pone.0283708 (PMC10042347; doi:10.1371/journal.pone.0283708)
Supplement: S1 Table — (DOCX) [file pone.0283708.s001.docx]

**Supplementary Table.**

|  | **Overall**  (n=900) | **Quantifiable RV Function+**  (n=450) | **Quantifiable RV Function-**  (n=450) | **p** |
| --- | --- | --- | --- | --- |
|  |  |  |  |  |
| **Demographic Indices** | | | | |
| Age (years) | 65.9 ± 15.9 | 66.9 ± 16.4 | 64.8 ± 15.4 | 0.04 |
| Male gender | 61% (552) | 59% (264) | 64% (288) | 0.10 |
|  | | | | |
| Hypertension | 63% (568) | 62% (280) | 64% (288) | 0.58 |
| Diabetes mellitus | 42% (382) | 44% (198) | 41% (184) | 0.35 |
| Obesity† | 29% (265) | 29% (127) | 31% (138) | 0.45 |
| Coronary artery disease‡ | 22% (198) | 24% (106) | 20% (92) | 0.26 |
| Tobacco use§ | 30% (273) | 28% (127) | 32% (146) | 0.17 |
| Heart Failure | 16% (143) | 17% (74) | 15% (69) | 0.64 |
| **Pulmonary Disease** |  |  |  |  |
| Asthma | 9% (78) | 10% (46) | 7% (32) | 0.10 |
| COPD | 7% (60) | 6% (29) | 7% (31) | 0.79 |
|  | | | | |
| ACE inhibitor/ARB | 31% (282) | 29% (131) | 34% (151) | 0.15 |
| Statin | 41% (368) | 42% (190) | 40% (178) | 0.42 |
| Beta blocker | 34% (302) | 38% (171) | 29% (131) | **0.005** |
| Aspirin | 27% (241) | 26% (116) | 28% (125) | 0.50 |

†Obesity was defined as BMI ≥ 30 kg/m^2^.

‡Coronary artery disease was defined as history of prior MI and/or coronary revascularization.

§Tobacco use indicated current and past smoking.
